# Analyzing and Modeling the Spread of SARS-CoV-2 Omicron Lineages BA.1 and BA.2, France, September 2021–February 2022

**DOI:** 10.3201/eid2807.220033

**Published:** 2022-07

**Authors:** Mircea T. Sofonea, Bénédicte Roquebert, Vincent Foulongne, David Morquin, Laura Verdurme, Sabine Trombert-Paolantoni, Mathilde Roussel, Jean-Christophe Bonetti, Judith Zerah, Stéphanie Haim-Boukobza, Samuel Alizon

**Affiliations:** Université de Montpellier, Montpellier, France (M.T. Sofonea);; Laboratoire Cerba, Saint Ouen L’Aumône, France (B. Roquebert, L. Verdurme, S. Trombert-Paolantoni, M. Roussel, S. Haim-Boukobza);; Centre Hospitalier Universitaire de Montpellier, Montpellier (V. Foulongne, D. Morquin);; Laboratoire Cerballiance Paris et Île-de-France Est, Paris, France (J.-C. Bonetti, J. Zerah);; Centre National de la Recherche Scientifique, Paris (S. Alizon)

**Keywords:** COVID-19, coronavirus disease, SARS-CoV-2, severe acute respiratory syndrome coronavirus 2, viruses, respiratory infections, zoonoses, vaccine-preventable diseases, epidemiology, mutation, PCR, genomics, mass screening, variants, France, Omicron, BA.1, BA.2

## Abstract

We analyzed 324,734 SARS-CoV-2 variant screening tests from France enriched with 16,973 whole-genome sequences sampled during September 1, 2021–February 28, 2022. Results showed the estimated growth advantage of the Omicron variant over the Delta variant to be 105% (95% CI 96%–114%) and that of the BA.2 lineage over the BA.1 lineage to be 49% (95% CI 44%–52%). Quantitative PCR cycle threshold values were consistent with an increased ability of Omicron to generate breakthrough infections. Epidemiologic modeling shows that, in spite of its decreased virulence, the Omicron variant can generate important critical COVID-19 activity in hospitals in France. The magnitude of the BA.2 wave in hospitals depends on the level of relaxing of control measures but remains lower than that of BA.1 in median scenarios.

The Omicron SARS-CoV-2 variant of concern (Pango lineage B.1.1.529, GISAID clade GR/484A) was detected in South Africa on November 26, 2021 (*1*). Rapid analyses demonstrated its increased transmissibility (C.A.B. Pearson et al., unpub. data, https://doi.org/10.1101/2021.12.19.21268038), high immune evasion potential (*2*,*3*), and low virulence (*4*–*6*) compared with the Delta variant. Furthermore, the biology of the virus appears to be different, having the potential to enter human cells through endocytosis and a pronounced tropism for the upper respiratory tract (*7**–**9*; T.P. Peacock et al., unpub. data, https://doi.org/10.1101/2021.12.31.474653; B.J. Willett et al., unpub. data, https://doi.org/10.1101/2022.01.03.21268111). After South Africa, the Omicron variant caused epidemic waves in many countries, including the United Kingdom (*10*), Denmark (*11*), and countries of North America (*12*).

The first Omicron lineage to dominate was BA.1 (B.1.1.529.1, Nextstrain clade 21K). However, in some countries, such as Denmark, its sister lineage BA.2 (former B.1.1.529.2, Nextstrain clade 21L) rapidly became dominant. BA.1 and BA.2 are highly divergent lineages (A.Z. Mykytyn et al., unpub. data, https://doi.org/10.1101/2022.02.23.481644), but their virulence and biology appear to be similar and the cross-immunity strong (M. Stegger et al., unpub. data, https://doi.org/10.1101/2022.02.19.22271112). Early reports suggest that BA.2 has a growth advantage over BA.1 (F.P. Lyngse et al., unpub. data, https://doi.org/10.1101/2022.01.28.22270044), possibly from a shorter generation time (i.e., average delay between consecutive infections in a transmission chain) (*10*).

Since January 2021, all the positive samples in France have been screened with variant-specific quantitative PCR (qPCR) assays targeting specific mutations (*13*). This close monitoring of the epidemic has low specificity, and the mutations targeted need to be updated to match the circulating variants, which is also why the monitoring is complemented by the whole-genome sequencing (WGS) of a subset of the samples.

We analyzed 324,734 variant-specific screening tests performed during September 1, 2021–February 28, 2022, in all 13 regions of mainland France. To understand lineage circulation, we generated SARS-CoV-2 whole-genome sequences for 16,973 of these samples (5.2%) over the same period. We analyzed the cycle threshold (Ct) values of the qPCR to gain further insights into the biology and epidemiology of the infections. Finally, we used these results to explore prospective scenarios regarding the dynamics of critical care unit (CCU) occupancy in France in 2022. This study was approved by the Institutional Review Board of the Centre Hospitalier Universitaire of Montpellier and is registered at ClinicalTrials.gov (identifier no. NCT04738331).

## Methods

### Cohort Description

The variant screening tests were performed by Cerba Laboratory (Saint Ouen L’Aumône, France) on samples that originated from partner centers in mainland France and tested positive for SARS-CoV-2 with a generic qPCR assay. Most of the samples originated from the general population (Tables 1, 2). We did not have access to additional details about patient symptoms; however, according to an earlier study on a similar cohort, nearly all the samples originated from nasopharyngeal swab specimens, and the proportion of symptomatic and asymptomatic individuals were comparable among the positive tests (*14*). To limit epidemiologic biases, we removed persons >80 years or <5 years of age from the dataset.

**Table 1:** Main characteristics of SARS-CoV-2 variant-specific screening tests (N = 131,478), France, September 1–December 18, 2021*

Characteristic	Value
Age of patient, y, median (95% CI)	36 (6–74)
Assay	
TIB Molbiol	4,887 (3.7)
PerkinElmer	33,037 (25.1)
ID Solutions (Evolution)	93,554 (71.2)
Context	
General population	127,337 (96.9)
Hospital	4,141 (3.1)
Region	
Ile-de-France	51,407 (39.1)
Hauts-de-France	16,938 (12.9)
Normandie	11,996 (9.1)
Nouvelle-Aquitaine	8,516 (6.5)
Provence-Alpes-Côte d’Azur	7,549 (5.7)
Occitanie	7,143 (5.4)
Corse	5,528 (4.2)
Bourgogne-Franche-Comté	5,155 (3.9)
Grand Est	5,136 (3.9)
Centre-Val de Loire	4,811 (3.7)
Bretagne	3,455 (2.6)
Other	1,296 (0.9)
Outcome	
A0B0C1	101,970 (77.6)
A0B0C0	6,969 (5.3)
A0B1C1	899 (0.68)
A1B0C1	37 (<0.1)
A1B0C0	15 (<0.1)
Other	21,588 (16.4)

*Values are no. (%) patients except as indicated.

**Table 2:** Results of ID Solution Revolution SARS-CoV-2 variant-specific screening tests (N = 193,256), France, December 18, 2021–February 28, 2022*

Characteristic	Value
Age of patient, y, median (95% CI)	36 (6–74)
Context	
General population	187,292 (96.9)
Hospital	5,964 (3.1)
Region	
Ile-de-France	40,185 (20.8)
Hauts-de-France	26,382 (13.7)
Normandie	31,205 (16.2)
Nouvelle-Aquitaine	13,236 (6.9)
Provence-Alpes-Côte d’Azur	31,299 (16.2)
Occitanie	9,034 (4.7)
Corse	8,031 (4.2)
Bourgogne-Franche-Comté	4,366 (2.3)
Grand Est	6,865 (3.6)
Centre-Val de Loire	10,412 (5.4)
Bretagne	9,405 (4.9)
Other	2,836 (1.5)
Outcome	
A0B9C1D0	12,955 (6.7)
A0B9C0D1	154,134 (79.8)
A0B9C1D1	173 (0.1)
A1B9C0D0	4,762 (2.5)
Other	21,232 (10.9)

*Values are no. (%) patients except as indicated.

### Variant-Specific Screening Tests

We first analyzed 131,478 screening tests performed during September 1–December 18, 2021. The assays used over this first period were ID SARS-CoV-2/VOC Evolution Pentaplex (ID Solutions, https://www.id-solutions.fr) (93,554 tests), VariantDetect (PerkinElmer, https://www.perkinelmer.com) (33,037 tests), and VirSNiP (TIB Molbiol, https://www.tib-molbiol.de) (4,887 tests). These tests targeted 3 mutations in the SARS-CoV-2 spike protein: E484K (mutation A), E484Q (mutation B), and L452R (mutation C). Denoting the absence of a mutation by a 0 and its presence by 1, A0B0C1 mostly corresponds to infections caused by the Delta variant, A0B0C0 to the Alpha or Omicron variant or an ancestral lineage, A0B1C1 to Kappa or Kappa-like variants, A1B0C0 to the Beta or the Gamma variant, and A1B0C1 to a Delta variant with an E484K mutation.

Because of the shift in variant frequencies, new screening assays were implemented in late 2021. We analyzed 193,256 tests performed during December 6, 2021–February 28, 2022, all using the assay ID SARS-CoV-2/VOC Revolution Pentaplex (ID Solutions). This assay still targeted mutations A and mutation C but also targeted S:K417N (mutation D). Denoting nontested mutations with a 9, then A0B9C1D0 most likely indicates infections caused by the Delta variant, A0B9C0D1 by the Omicron variant, A1B9C0D0 by the Gamma variant, A1B9C0D1 by the Beta variant, and A0B9C0D0 by the Alpha variant or the B.1.640 lineage. Finally, A0B9C1D1 can either indicate an infection by Delta with a 417N mutation, Omicron with a 452R mutation, or a Delta–Omicron co-infection.

For the ID Solutions Pentaplex tests, we analyzed 4 Ct values. Three of these values correspond to primers targeting the mutations of interest: S:417N, S:452R, or S:484K, the last to a primer targeting the nucleoprotein gene, which was used as a control.

### Whole-Genome Sequencing

Next-generation sequencing (NGS) was performed by Cerba Laboratory for 16,973 samples with a Ct <30 using the CovidSeq amplicon-based NGS assay according to supplier recommendations (Illumina, https://www.illumina.com) and after a Janus/Chemagic RNA extraction (Perkin Elmer) from the nasopharyngeal swab. All sequences obtained were submitted to the EMERGEN Consortium Database (Santé Publique France, https://www.santepubliquefrance.fr/dossiers/coronavirus-covid-19/consortium-emergen) and GISAID (https://www.gisaid.org).

### Statistical Analyses

#### Multinomial log-Linear Model

We performed a multinomial log-linear model with the formula variant = ꞵ_0_ + ꞵ_1_ age + ꞵ_2_ assay + ꞵ_3_ location_sampling + ꞵ_4_ date:region + ε, where the ꞵ_i_ are the model parameters, ε the residuals, and the variable age is the individual age (treated as an integer and centered and scaled), location_sampling is a binary variable indicating whether the sample was collected in a hospital or in the general population, assay is the qPCR assay used, date is the sampling date (treated as an integer and centered and scaled), and region is the administrative region of residency in France. We included interactions between region and sampling date to detect temporal trends.

To make results easier to interpret, we computed relative risk ratios (RRRs). These ratios reflect, for a given variable, the risk for belonging to 1 of the outcomes (variant detection in this study) compared with the control group.

#### Growth Advantage Calculation

**Figure 8:**
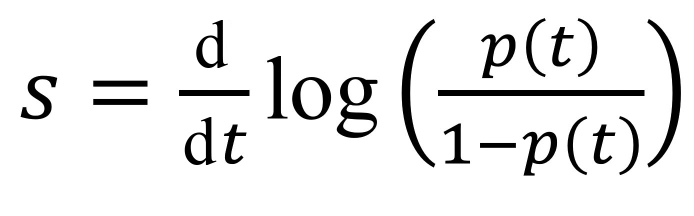
Growth Advantage Calculation Equation

We computed growth advantages by using earlier methods based on Malthusian population growth rates (*15*–*18*). If we denote by *p*(*t*) the frequency of an allele of interest (e.g., A0B0C0 test results) in the population (e.g., A0B0C0 and A0B0C1 test result), then the selection coefficient corresponds to the following rate (Figure 8):

This value is the inverse of a duration, and comparing it to earlier estimates requires a scaling for the generation time, the mean of which, T, is approximated by the mean serial interval (*19*). Overall, the growth advantage of a variant (e.g., A0B0C0) over another (e.g., A0B0C1) scaled for 1 infection generation is denoted as sT and given by the formula s_T_ = s × T. We estimated s_T_ by using the fitted values from a generalized linear model with a logit link to control for the covariates listed.

We used 21-day windows to estimate growth advantage, which corresponds to >4 generations of infection given the average generation time used. This number was chosen to be able to detect potential signals, while still obtaining a good temporal resolution of the estimated.

#### Ct Values Linear Modeling

We used a linear model with the following formula: Ct =Ɣ_0_ + Ɣ_1_ age + Ɣ_2_ variant + Ɣ_3_ location_sampling + Ɣ_4_ date × region + ε, where the Ɣ_i_ indicate the model parameters, ε the residuals, and the covariates are the same as in the multinomial model. The variant was determined either by reverse transcription qPCR or WGS. The sampling date was included in the model because growing epidemics can be associated with lower Ct values than declining epidemics (*14*,*20*).

Using a likelihood ratio test, we showed that the presence of the variant covariate does improve the model. We assessed covariate significance by using an analysis of variance (ANOVA) with a type II error using the ANOVA function from the companion to applied regression package in R (R Project for Statistical Computing, https://www.r-project.org). We computed estimated marginal means for the Ct values associated with the screening tests results by using the emmeans function from the eponym R package. We plotted the fitted values from the linear model by using the predict function in R. The statistical methods are further described (Appendix 1), and raw data and R scripts are available online (https://doi.org/10.5281/zenodo.6536220).

#### Epidemiologic Modeling

**Figure 1:**
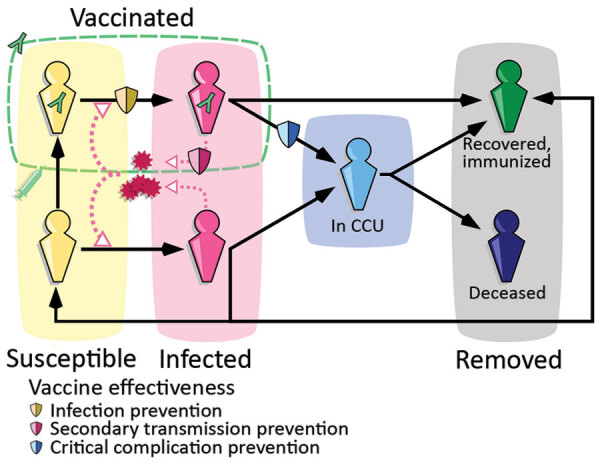
Epidemiologic modeling of the SARS-CoV-2 Omicron BA.2 wave dynamics, France. Simplified flowchart of the Covidsim framework. Persons can move between several compartments in the general population (in yellow or pink depending on the infection status), in CCUs in blue and removed from the system, either because of their immunity to BA.2 or of death (in gray). Part of the general population is vaccinated (green dashed line), which affects epidemiologic dynamics in 3 ways (illustrated with the shields), namely reduced infectivity, reduced virulence, and reduced risk for infection. CCU, critical care unit.

We used the previously developed framework Covidsim, which accurately captures the national CCU admissions for SARS-CoV-2 in France and the associated mortality incidence time series (*21*). The underlying model is deterministic, is structured in discrete time, and uses CCU incidence and prevalence data, as well as mortality data, to estimate parameters of interest (Figure 1, Appendix 1). A retrospective analysis showed its ability to provide robust projections up to 5 weeks ahead (*22*).

In the model, the number of vaccinated persons followed the national campaign in France (Système d’Information VAccin Covid data) and the number of persons with postinfectious immunity results from the model’s reconstruction of the epidemic. The protection against infection and severe illness depends on the type of immunity (vaccine [*23*] or postinfectious [*24*]) and the variant. These values, like others, were informed from literature data, technical reports, and preliminary work.

Having a mechanistic model enables us to explore prospective scenarios for CCU activity. We did so by formulating assumptions regarding the intensity of future control measures and incorporating our estimates of growth advantage and relative frequency of the variants into the model.

In this study, the temporal reproduction number (Rt) corresponds to the average number of secondary infections caused by an infected person at date t and is estimated by using national hospital admission data (https://www.data.gouv.fr/fr/datasets/donnees-hospitalieres-relatives-a-lepidemie-de-covid-19) and the EpiEstim method (*25*). We shifted the dates in the incidence time series to compute Rt, setting the median time between infection and CCU admission to 14 days (*21*,*26*).

## Results

### A0B0C0 Emergence

**Figure 2:**
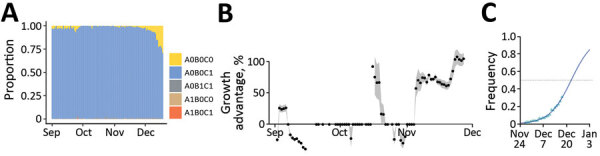
Monitoring and quantifying variant spread in using SARS-CoV-2 variant-specific screening tests (N = 103,757), France, October 1–December 18, 2021. A) Raw proportion of the test outcomes. B) Growth advantage of A0B0C0 tests over A0B0C1 in France. Points indicate the median growth advantage estimated on a 21-day sliding window; the gray shading indicates 95% CI. C) Estimated frequency and growth advantage of A0B0C0 relative to the sum of A0B0C0 and A0B0C1 tests in France, corresponding to the last point of panel A. Triangles show the fitted values from the model, the line the model output, and the gray shading the 95% CI. Raw occurrence data from panel A is stratified by region in Appendix 1 Figure 1. Test designations indicate the absence of a mutation by a 0 and its presence by 1; the mutations are S:484K (A), S:E484Q (B), and S:452R (C); A0B0C1 mostly corresponds to Delta variant, A0B0C0 to Alpha or Omicron variant or an ancestral lineage, A0B1C1 to Kappa or Kappa-like variants, A1B0C0 to Beta or the Gamma variant, and A1B0C1 to a Delta variant with an E484K mutation.

We first analyzed variant-specific screening tests collected during September 1–December 18, 2021 (Figure 2, panel A). Most of these tests originated from the general population (96.6%) and showed coverage differences between regions of France (Table 1). The most common assay used (71%) was that from ID Solutions (Evolution). The raw number of tests performed follows the incidence curve of the epidemic (Appendix 1 Figure 1).

Focusing on the tests performed during October 25–December 18, 2021 (i.e., when the epidemic was increasing), we used a multinomial regression model to identify covariates associated with the test outcome (Table 3). A0B0C0 infections (consistent with Omicron) were found in younger persons than were A0B0C1 infections (consistent with Delta); RRR was 0.85 (95% CI 0.83–0.88) per age unit (equal to 56 years in this study). We also detected strong temporal increases in most of the regions of France that had RRR >10 per day. In some regions, we detected a temporal increase of A0B1C1 tests, consistent with the Kappa variant. Finally, in our dataset, the (rare) A1B0C1 tests only showed a slight temporal increase in 2 regions (Bretagne and Hauts-de-France).

**Table 3:** Relative risk ratios of covariates associated with SARS-CoV-2 variant-specific screening tests (N = 103,757), France, October 1–December 18, 2021*

Covariate	Relative risk ratio (95% CI)				
	A0B0C0	A0B1C1	A1B0C0	A1B0C1	Other
Intercept	0 (0.00–0.01)	0.01 (0–01)	NS (0–0)	NS (0–0)	0.18 (0.17–0.18)
Age, scaled†	0.85 (0.83–0.88)	1.08 (1.0–1.2)	NS (0.7–2.4)	NS (0.5–1)	0.82 (0.8–0.83)
Context					
General population	Referent	Referent	Referent	Referent	Referent
Hospital	NS (0.82–1.1)	0.37 (0.2–0.69)	NS —	NS —	0.88 (0.79–0.99)
Assay					
ID Solutions	Referent	Referent	Referent	Referent	Referent
PerkinElmer	2.0 (1.8–2.1)	0.46 (0.38–0.56)	NS (0–3.8)	NS (0.1–1.1)	0.82 (078–0.85)
TIB Molbiol	2.1 (1.6–2.6)	10.9 (9–13)	NS (0.9–23)	8.3 (3.1–22)	1.94 (1.8–2.1)
Date and region					
Ile-de-France	87.0 (75–100)	4.4 (3.4–5.7)	NS (0–7.5)	NS (0.3–6.5)	1.7 (1.6–1.8)
Bourgogne-Franche-Comté	10.5 (7.8–14)	8.3 (5.6–12)	NS (no values)	NS (0–49)	0.63 (0.53–0.74)
Bretagne	37.6 (28–51)	NS (0.91–5.4)	NS (no values)	21.6 (2–200)	1.3 (1.1–1.6)
Centre-Val de Loire	46.1 (37–57)	NA (0.8–3.5)	NS (0–370)	NS (0–98)	NS (0–0)
Corse	86.4 (71–100)	0.2 (0.05–0.5)	NS (0–310)	NS (0–56)	1.9 (1.7–2.2)
Grand Est	22.2 (18–28)	3.7 (2.3–5.8)	NS (0.5–80)	NS (0–100)	0.49 (0.42–0.58)
Hauts-de-France	44.8 (38–53)	NS (0.4–1.2)	NS (0–10)	18.0 (5.5–58)	1.17 (1.10–1.30)
Normandie	38.2 (32–46)	2.2 (1.4–3.4)	NS (0–23)	NS (0–15)	0.77 (0.69–0.86)
Nouvelle-Aquitaine	17.6 (14–22)	2.7 (1.7–4.4)	NS (0–51)	NS (0–16)	0.43 (0.37–0.50)
Occitanie	19.8 (16–25)	7.7 (5.3–11)	NS (0–95)	NS (0–31)	NS (0.82–1.1)
Provence-Alpes-Côte d’Azur	19.5 (16–25)	NS (0.6–2.2)	NS (0–320)	NS (0–67)	0.62 (0.54–0.71)
Other	37.6 (26–54)	NS (0.6–6.7)	NS (no values)	NS (no values)	NS (0.63–1.10)

*Model only analyzes tests performed after October 25, 2021; tests performed before that date are described in Appendix 1 Table 1. NS, not significant. †Age variable is centered and scaled (1 scaled unit corresponds to 56 years).

We then estimated growth advantages of A0B0C0 over A0B0C1 infections during 21-day time windows. The advantage was adjusted for covariates and assumed to be constant over each window. In September 2021, A0B0C0 infections were spreading less rapidly than A0B0C1 (Figure 2, panel B). This finding is consistent with the rapid increase of the Delta variant at the time (*18*). The pattern shifted at the end of November, with a 50% growth advantage of A0B0C0 infections, which increased to 105% (95% CI 96.1%–114%) in the last time window. According to this model, A0B0C0 infections became more frequent than A0B0C1 infections during the week of December 20 (Figure 2, panel C), with strong variations across regions (Appendix 1 Figure 1).

### The A0B9C0D1/Omicron Wave

**Figure 3:**
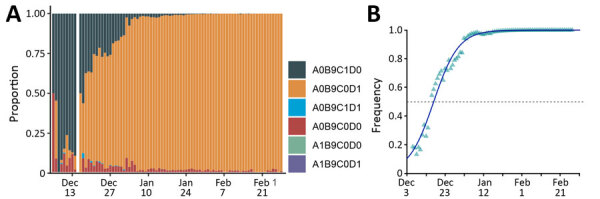
Monitoring and quantifying variant spread using ID Solutions Revolution tests (N = 193,256), France, December 6, 2021–February 28, 2022. A) Raw proportion of the test outcomes. B) Estimated frequency of A0B9C0D1 relative to the sum of A0B9C0D1 and A0B9C1D0 tests in France. Raw occurrence data from panel A is stratified by region in Appendix 1 Figure ). Test designations indicate the absence of a mutation by a 0 and its presence by 1 (9 means the mutation was not tested); mutations are the same as in Figure 2 and D is S:417N; A0B9C0D1 mostly corresponds to Omicron variant, A0B9C1D0 to Delta variant and A0B9C1D1 to Omicron-Delta coinfection.

The new screening test targeting the K417N mutation enabled us to better document the spread of the Omicron variant (Table 2). In December 2021, the A0B0C0 wave was mainly caused by viruses bearing the K417N mutation (Figure 3, panel A). Furthermore, the proportion of A0B0C0 tests not attributable to Omicron decreased toward the end of the year. Finally, we also noted potential co-infections of Omicron and Delta in December.

We then estimated the growth advantage of A0B9C0D1 over A0B9C1D0 during December 6, 2021–February 28, 2022 (Figure 3, panel B). The resulting estimate (96.5% [95% CI 87.9%–105%]) is very consistent with the results obtained using a less-specific test on the early stages of the wave.

**Figure 4:**
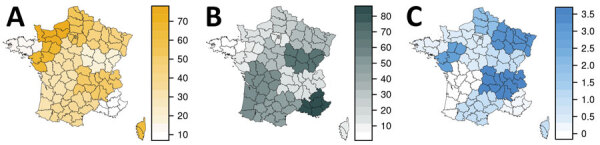
Frequency of A0B9C0D1 (A), A0B9C1D0 (B), and A0B9C1D1 (C) SARS-CoV-2 variant test results in mainland regions of France during week 51 of 2021. The colors show the prevalences (in percentages), which are corrected for covariates (age and sampling context). Includes 7,166 tests of the tests shown in Figure 3 but performed December 20–26, 2021. Test designation is the same as in Figure 3.

We observed a shift between the Omicron waves in the different regions of France (Figure 4). For instance, in the South-East area, Delta was still dominant during week 51 of 2021. As expected, we also saw that tests consistent with co-infections of Omicron and Delta were more frequent in regions where the 2 variants were cocirculating in substantial frequencies. This shift in different regions can explain the second increase in growth advantage of A0B0C0 tests observed in November (Figure 2, panel C).

### Sequencing Reveals a Shift from BA.1 to BA.2

**Figure 5:**
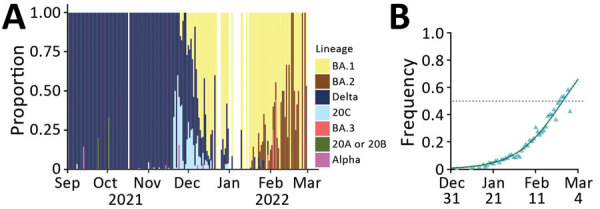
Monitoring and quantifying SARS-CoV-2 variant spread using whole-genome sequencing, France. A) Raw proportion of SARS-CoV-2 lineages inferred from whole-genome sequences of 16,973 samples. B) Estimated proportion and growth advantage of the BA.2 variant with respect to the BA.1 variant. Raw occurrence data from panel A is stratified by region in Appendix 1 Figure).

Because variant screening tests only target 3 mutations, we analyzed whole-genome sequences of ≈5% of the positive samples (Figure 5, panel A). This analysis revealed that before October 2021, A0B0C0 tests mostly originated from Delta variant infections, whereas in November they originated from rare lineages or from the 20C lineage. A more precise analysis shows that these mostly correspond to the B.1.640 lineage. Beginning near the end of November, half of these tests were associated with the Omicron variant; this percentage increased to >80% during December.

Beginning in the second week of January 2022, some of the screening outcomes consistent with Omicron (A0B9C0D1) were associated with the BA.2 variant (Figure 5, panel B). This proportion increased over the next several weeks. Using the sequencing data, we estimated a growth advantage of BA.2 over the BA.1 Omicron lineage of 48.9% (95% CI 44.2%–53.6%). BA.2 accounted for most variants at the end of February, meaning that the Omicron variant BA.1 lineage only dominated the epidemic in France for <3 months (Figure 5, panel B).

### Ct Differences

**Figure 6:**
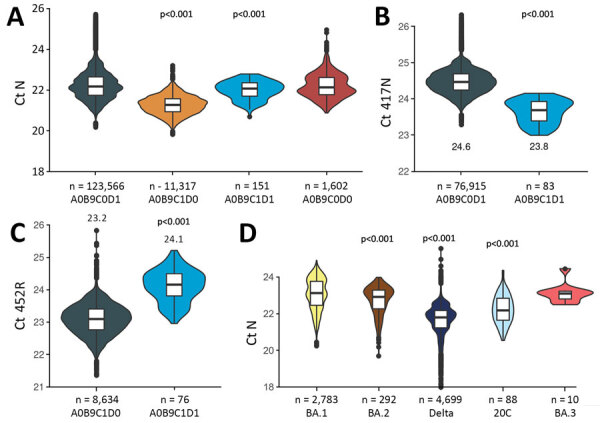
Ct values from the SARS-CoV-2 variant-specific screening quantitative PCR tests (N = 136,636), France, December 6, 2021–February 28, 2022. Ct values refer to the control (nucleoprotein) gene (A and D), 417N mutation (B), and 452R mutation (C). Values are shown as a function of the test outcome (A, B, and C) or the virus lineage (D). P values derived from a t-test where the reference variable is either A0B9C0D1 or BA.1. Boxes within violin plots show the median (horizontal line within box), 50% (box tops and bottoms), and 95% CIs (error bars). Tests were the same as in Figure 3, but only screening tests with Ct <28 were included to ensure robust screening results. Ct, cycle threshold; N, nucleoprotein gene.

For the tests performed during December 16, 2021–February 28, 2022, we used a linear model to explore differences in Ct values between variants. All the covariates were significant according to ANOVA with a type II error (Appendix 1 Table 1). Ct values tended to decrease with age or to be lower in samples from hospitals (Appendix 1 Table 2), which is consistent with earlier results (*14*). Furthermore, A0B9C0D1 tests exhibited significantly higher Ct values than A0B9C1D0; fitted median values were 22.1 versus 21.4 (Figure 6, panel A). This result suggests lower amounts of genetic material in the samples.

To further investigate these patterns, we analyzed the Ct values of the mutations targeted by the assay. We found that the Ct for the 417N mutation was higher in single infections (A0B9C0D1) than in co-infections (A0B9C1D1) (Figure 6, panel B). This finding is consistent with the greater ability of Omicron compared with Delta to infect immunized hosts, assuming that such breakthrough infections have a lower virus load (*27*,*28*). For the 452R mutation, we found the opposite pattern (Figure 6, panel C).

Finally, we analyzed the Ct values of the control gene as a function of the virus lineage inferred from the NGS data (Figure 6, panel D). BA.1 samples had higher Ct values than did Delta samples. Furthermore, BA.2 samples had lower Ct values than did BA.1 samples.

### Modeling Scenarios

On December 22, 2021, we incorporated the inferred growth advantage of Omicron/BA.1 over Delta into Covidsim (*21*) to explore an optimistic and a pessimistic scenario running through mid-March 2022. These scenarios differed in terms of the assumptions made regarding the reduction of Omicron virulence compared with Delta (3-fold vs. 2-fold) and vaccine protection against infection (75% vs. 40%) and severe illness (95% vs. 80%). Even though our assumption that the epidemic was under control at the end of 2021 was too optimistic, both the optimistic and the pessimistic scenarios showed that CCU activity was likely to remain high over January and February 2022, which proved to be accurate (Appendix 1 Figure 4).

**Figure 7:**
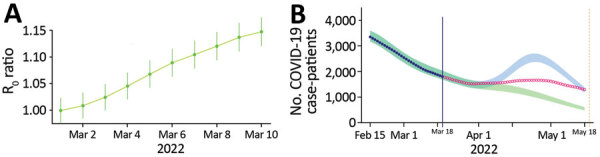
Analyzing and modeling the SARS-CoV-2 Omicron BA.2 epidemic wave in France. A) Ratio between the predicted and observed reproduction number (R0) based on BA.2 frequency and growth advantage. B) National critical care bed occupancy in 2 scenarios depending on baseline transmission increase. CIs are calculated from that of the frequency and growth advantage of BA.2 (Figure 5, panel B). The vertical blue line indicates the day the model was performed, the dark blue dots the data, and the shaded areas the 95% range of the model simulations. The 2 scenarios differ according to the capping of the increase of the baseline transmission rate, mimicking either a limited (green) or a strong (blue) easing preventive measures in March 2022 in France. Red open circles indicate data collected after the scenarios were modeled (i.e., not used in the inference or the modeling). The vertical yellow line indicated the last day the data were collected for the figure. Appendix 1 further details model.

Given our estimations of the frequency of the Omicron/BA.2 sublineage in the population and its growth advantage over BA.1, we can predict the temporal increase of the epidemic Rt. We compared this predicted Rt with that calculated for the period March 1–10, 2022, using national hospital admission data, and found that from March 3 the ratio between the 2 was greater than unity (Figure 7, panel A). This result suggests that the epidemic growth cannot solely be explained by variant replacement and involves other drivers (e.g., the end of the holiday periods in some regions starting February 21, 2022).

Finally, on March 17, 2022, by using consolidated estimates of relative virulence (*6*) and vaccine effectiveness (*23*) for Omicron variants, we explored 2 prospective scenarios for nationwide COVID-19 CCU activity depending on the intensity of the relaxation of the control over the epidemic: Rt at the peak as 1.1 or 1.6 (Figure 7, panel B). We found that a new hospital peak was possible in the more pessimistic case, but its height remained below half of the peak experienced during the first Omicron wave in January.

## Discussion

Variant-specific qPCR represents a flexible and cost-efficient surveillance method to obtain timely descriptions of SARS-CoV-2 epidemics. Thanks to a dense follow-up, we estimated that the Omicron variant spread in France with a 2-fold growth advantage over Delta (i.e., higher than that recorded for the Delta variant vs. the Alpha variant in June 2021) (*18*). This finding is consistent with estimates from South Africa (C.A.B. Pearson et al., unpub. data) and the United Kingdom (S. Abbott, et al., unpub. data, https://doi.org/10.1101/2022.01.08.22268920). Some estimates from Denmark suggest even higher advantages but using a different method (relying on reproduction numbers and not growth rates) and GISAID genomic data, which means a lower coverage and potentially strong reporting delays (*29*).

Thanks to the WGS of 5% of the samples, we were able to confirm the nature of the variants spreading and to detect a replacement of the BA.1 Omicron lineage by the BA.2 with a growth advantage of ≈50% (the precise value depends on the serial interval used [19]). This finding is consistent with the qualitative trends reported from South Africa (*30*) and the United Kingdom (*10*) and from household data in Denmark (F.P. Lyngse et al., unpub. data). Note that these estimates tend to rely on the spike gene target failure, which is observed in a ThermoFisher assay for Omicron/BA.1 but not for Delta and not for BA.2. In our study, using variant-specific screening tests designed to target 3 specific mutations conferred a greater specificity of the results.

By analyzing qPCR Ct values, we found that samples from BA.1/Omicron infections had significantly higher Ct values than those from Delta infections. Although care must be taken when analyzing Ct values, especially for coronaviruses (*31*), this finding suggests a lower amount of virus genetic material in the samples. This result is intriguing given the large growth advantage of Omicron over Delta. A possible interpretation is that the Omicron variant is more prone to infecting immunized hosts (*2*,*3*) and, in vaccinated hosts, such breakthrough infections have been reported to have lower virus load than infections of nonvaccinated hosts (*27*,*28*).

We did not have access to the vaccination status of the persons from whom samples were taken. However, a potential overrepresentation of immunized hosts among Omicron infections is consistent with the lower values for the Ct associated with the 417N mutation in Delta–Omicron co-infections compared with Omicron monoinfections. Because Delta is less prone to immune evasion than Omicron, we expect the proportion of immunized hosts to be low in co-infections.

A limitation of our approach is that we cannot readily identify the origin of the growth advantage of BA.2 with respect to BA.1. This advantage could be caused by a shorter generation time for BA.2 infections (*10*), which is consistent with our finding that BA.2 samples have lower Ct values than those for BA.1 samples. Furthermore, although we do control for the sampling date as a covariate, this difference could reflect the epidemic trend given that Ct values are expected to be lower in expanding epidemics (*14*,*20*).

Our study highlights both the strengths and weaknesses of variant-specific screening assays (also sometimes called allele-specific reverse transcription qPCR). The advantage is that these assays enable rapid detection of variant replacement (we could detect a signal in the A0B0C0 tests in early December, at a time when the Omicron frequency <5%). However, the information about the circulating lineage is limited and, for example, the onset of the BA.2 wave in France could only be detected by using sequencing data. Furthermore, test interpretations vary with time. Before September 2020, some A0B0C0 tests were caused by the Alpha variant and by the Delta variant with a low Ct. In late October, before being associated with Omicron infections, most of these tests were probably attributable to lineage B.1.640, first detected in the Democratic Republic of the Congo (*32*). Temporal variations (Figure 2, panel B) may also originate from spatial heterogeneity; growth advantages are calculated for large administrative units, and variant epidemics can be at different stages in different regions. Finally, delays in data reporting can matter in the initial stages of variant epidemics.

Beyond nowcasting (near–real-time estimating) variant replacement rates, epidemiologic models represent a powerful tool to explore prospective scenarios. By combining our estimates of growth advantage with literature data, especially on vaccine protection, we showed that the decrease in Omicron virulence (*6*) was not sufficient to allow for a steep decrease in critical COVID-19 activity in hospitals in France >1 month before the reported incidence peak, hence helping CCU to anticipate the number of beds necessary and plan for the return to regular activity for the other hospital sectors.

Appendix 1 Additional information about analyzing and modeling the spread of SARS-CoV-2 Omicron lineages BA.1 and BA.2, France, September 2021–February 2022.

Appendix 2 Appendix 3. Members of the EMERGEN Consortium.
