# Supplementary material for: Analyzing and Modeling the Spread of SARS-CoV-2 Omicron Lineages BA.1 and BA.2, France, September 2021–February 2022
Source: Emerg Infect Dis. 2022 Jul;28(7):1355–65. doi: 10.3201/eid2807.220033 (PMC9239895; doi:10.3201/eid2807.220033)
Supplement: Appendix 1 — Additional information about analyzing and modeling the spread of SARS-CoV-2 Omicron lineages BA.1 and BA.2, France, September 2021–February 2022. [file 22-0033-Techapp-s1.pdf]

Article DOI: https://doi.org/10.3201/eid2807.220033

Analyzing and Modeling the Spread of SARS-CoV-2 Omicron Lineages BA.1 and BA.2, France, September 2021–February 2022

Appendix 1

Multinomial Log-Linear Model

To perform the multinomial log-linear model, we used the multinom function from the nnet R package. This function uses neural networks to perform model selection in a stepwise manner starting from the null model (i.e., without any predictor).

The model formula was the following: variant ~ age + assay + location_sampling + date:region, where age is the age of the individual (which is treated as an integer and centered and scaled), location_sampling is a binary variable indicating whether the sample was collected in a hospital or not, assay corresponds to the type of screening test used, date is the sampling date (which is treated as an integer and centered and scaled), and region is the French administrative region of residency.

The multinom function uses an AIC criterion to identify the best model and returns the estimated multinomial logistic regression coefficients as well as their standard error (SE).

These can be used to calculate a z-test statistic, which is simply the ratio of the coefficient value to the SE. From there, we can construct a p-value, $p > |z|$, which is the probability the z-test statistic would be observed under the null hypothesis and assuming that z follows a normal distribution. Here, we use a classical significance threshold of $\alpha = 5\%$. When the p-value is smaller than α, the null hypothesis can be rejected and the parameter is considered to be significant.

Note that an alternative approach could be to calculate the 95% confidence interval for the coefficient value of the multinomial model using the SE and a critical value on the standard normal distribution.

To give a more intuitive interpretation of the results, we compute the relative risk ratios (RRR) by taking the exponential of the coefficient values of the model. The RRR reflects, for a given variable, how the risk of belonging to one of the outcomes (here variant detection) varies compared to the control group.

Further details about multinomial log-linear models and their interpretations can be found at https://stats.idre.ucla.edu/stata/output/multinomial-logistic-regression/.

Selection Advantage Estimation

The selection advantage is estimated from the Malthusian growth rate of the 'population' of variant-specific tests. More precisely, following methods developed in population genetics to estimate the selection coefficient of a mutant allele compared to a wild type allele (*1*), and following earlier studies in epidemiology (*2–4*), we calculate the selection coefficient s by fitting a logistic growth model to the time series of variant frequency.

Indeed, provided that the selection coefficient s does not var.y over time, and by denoting p(t) the frequency of an allele (here screening test result A0B0C0 or A0B0C1) in the population (here all tests with results A0B0C0 or A0B0C1), we have the following relationship:

$$s = \frac{d}{dt} log\left(\frac{p(t)}{1-p(t)}\right) \text{ (S1)}$$

Note that this value needs to be scaled with respect to the generation time T, which is here obtained from the serial interval calculated by Nishiura et al. (*5*). Overall, the transmission advantage sT of A0B0C0 tests over A0B0C1 tests is given by the formula sT = s T.

To estimate s, for each region of interest separately, we first perform a generalized linear model (GLM) with a binomial distribution of the residuals (i.e., a logistic regression) where the response variable is the test result (A0B0C0 or A0B0C1) and the covariates are the age of the individual (which is treated as an integer and centered and scaled), the assay used for the test, the sampling date (which is treated as an integer and centered and scaled), and the sampling region,

which is the French administrative region. We then use the fitted values from the GLM to perform the fit of the logistic growth function.

We use 21 days windows to estimate transmission advantage, which corresponds to two 95% confidence intervals (CI) of the serial interval used. This duration was chosen to be long enough to avoid false-positive signals and short-enough to allow for rapid detection of variations in s.

We also inferred the transmission advantage using the updated version of the R package EpiEstim (*6*) available at https://github.com/mrc-ide/EpiEstim. We only consider A0B0C0 and A1B1C1 test results and assume that both have the same serial interval (that from [*5*]).

Further details about the implementation of the inference can be found in the Supplementary R script with the Supplementary data.

Cycle Threshold Analyses

Statistically, we use a linear model with the following formula:
Ct ~ age + variant + location_sampling + date*region, where age is the age of the individual (which is treated as an integer and centered and scaled), variant is the outcome of the screening test, location_sampling is a binary variable indicating whether the sample was collected in a hospital or not, date is the sampling date (which is treated as an integer and centered and scaled), and region is the French administrative region of residency. We also include interactions between the region and the sampling date in the model to capture variations in Ct values that could be linked to differences in epidemic reproduction numbers. Indeed, growing epidemics can be associated with lower Ct values than declining epidemics (*7*,*8*).

We used a likelihood ratio test to compare this model and a model without the variant covariate. Adding this covariate does significantly improved the model.

Covariate significance was assessed using an analysis of variance (ANOVA) with a type II error using the Anova function from the car package in R.

The estimated marginal means (EMMs) for the Ct values associated with the screening tests results were computed using the emmeans function from the eponym R package.

Mathematical Modeling

The structure of the mathematical framework used is shown in Figure 6, panel A in the main text. Each age class is split into compartments (depicted by the figurines in the chart) according to their infectious, clinical and immunological statuses. Susceptible individuals (in yellow) can be infected if exposed to the SARS-CoV-2 by infected individuals (in pink) in the community (note that aged care facilities are not included). A fraction of the infected individuals develop a critical COVID-19 form, defined as requiring critical care and/or leading to hospital death (for simplicity, only patients admitted in critical care are depicted on the flowchart). Vaccination is implemented following the VAC-SI time series (data from Santé Publique France) and assumed to reduce the probability of three events, namely being infected if exposed, transmitting the virus if infected and developing critical complications if infected. Additionally, we assume that all individuals having recovered from a post-vaccine infection are immunized, contrary to unvaccinated individuals a fraction of which can become infected again (*9*). Formal and parameterization details are provided in (*10*), the system of which was updated according to the flowchart in Figure 6, panel A.

The COVIDSIM Framework

The main structure of COVIDSIM has been described in details in an earlier study (*10*).

In short, the underlying model is structured in discrete time and uses COVID-19 critical care unit (CCU) incidence and prevalence data, as well as mortality data, to estimate key parameters of the epidemic in France. Individuals can move between compartments that var.y in terms of infectivity (Figure 6, panel A in the main text). The transition between compartments depends on the time spent in each compartment meaning that the model is non-Markovian (i.e., it captures ‘memory’ effects).

For simplicity, the transmission model is not stratified by age. However, the probability of developing critical forms, being admitted to critical care, and dying from COVID-19 is age-stratified according to published data (*11*). In general, the model parameters correspond to the French epidemic: their initial values are derived from literature data, technical reports or preliminary work, and are then regularly adjusted to reflect hospital dynamics (*12*).

Vaccination follows the French national campaign data (VAC-SI data from Santé publique France, available at https://www.data.gouv.fr) with simplifying assumptions. In particular, we assume that all vaccines act as a 1-dose vaccine, using the date of the second dose as the date of vaccination. The proportion of people already infected is the result of the model's reconstruction of the epidemic.

The level of protection of the hosts depends on the type of protection. Natural post-infection is assumed to confer full protection to 85% of the individual, the 15% others remaining immunologically naïve. For vaccine immunity, we distinguish between different types of protection. In the most optimistic scenarios, we assume protection of 80% against infection and of 95% against the occurrence of severe forms if infected. Furthermore, based on our earlier investigations of the French epidemic (M.T. Sofonea et al., unpub. data, https://osf.io/6ebxu), we assume that the drop in infectiousness in so-called breakthrough infections in vaccinated individuals is 50%.

Regarding the Omicron wave more specifically, our biologic knowledge improved over the duration of this study. The first modeling work was performed on December 22, 2021, at a time when the vaccine evasion and severity properties of Omicron were poorly known. The second modeling work was performed on 17 March 2022 and we were able to lean on more detailed data regarding the differences between Delta and Omicron in terms of severity (*13*) and vaccine protection (*14*). The assumptions made in these two works a further described below.

Importantly, given the timescales considered, we neglect immune waning in the model, which means that on a long time scale our estimates could be over-optimistic. We also do not include seasonal variations, which have been shown to explain approx. 20% of the variance in reproduction number Rt (*15*).

Late 2021 Scenarios: The BA.1 Wave

The goal of this first model, which was performed on December 22, 2021, was to explore the impact of the Omicron variant on national CCU activity. This model was not intended to predict the future but rather to generate trends under assumptions that are arbitrarily optimistic for the most part. In particular, we made the following assumptions:

- The set of people vaccinated is homogeneous (e.g., no stratification according to the number of doses), which can be interpreted as assuming that the 3rd dose is rapidly generalized to the whole population.
- There is no decrease in immune protection, i.e., waning, over time.
- The proportion of previously infected people that Omicron can reinfect is identical to that of Delta (i.e., 15% [*9*]).
- The generation time (number of days between the moment a person is infected and the moment he/she infects another person) is the same for all the variants.
- The duration of stay in critical care remains unchanged.
- There are no ’New Year’s Eve’, ’holiday’, ’back-to-school’ or meteorological effects on the epidemic spread.
- The epidemic reproduction number (Rt) grows from 1.08 on December 23, 2021, to 1.25 on December 28, and to 1.5 on January 7, 2022, which corresponds to a 10% decrease in the reproduction number at the peak compared to what would be expected by extrapolating the estimated Omicron growth from the screening data at the time.
- From 15 January onward, a slowdown in the epidemic occurs due to public health interventions, spontaneous behavioral changes, and/or natural saturation related to spatial structure with a drop in Rt to 0.95.

In this model, we also incorporated the growth advantage of Omicron/BA.1 versus Delta and the estimated frequency of Omicron/BA.1 in the population estimated from the variant-specific screening test data (Figure 2, panel C in the main text). Since COVIDSIM is a single-strain model, the replacement of Delta by Omicron was modeled by adjusting the transmission rate, immune protection, and severity parameters as a function of the increase in the proportion of Omicron infections.

We then simulated two scenarios that differed in their assumptions regarding the differences between Omicron and Delta in terms of vaccine protection and virulence:

1. An “optimistic” scenario, with a 3-fold reduction in the probability of developing a critical form compared to Delta, a 75% vaccine protection against infection, and 95% protection against critical forms (therefore, an excellent effect of the 3rd dose).
2. A “pessimistic” scenario, where the probability of developing a critical form was only divided by 2 compared to Delta, and where the vaccine protection was only 40% against infection and 80% against critical forms.

Most of the assumptions made here, e.g., that on January 15, 2022, the Omicron epidemic would be decreasing, were rather optimistic. Therefore, the goal of these scenarios was to provide a lower bound. In other words, we wanted to estimate the minimal consequences on CCU of the Omicron variant wave.

The results of the model are shown in Appendix Figure 4.

2022 Scenarios: The BA.2 Wave

Detailed data about the Omicron/BA.1 was rapidly available and allowed us to carefully parameterize our COVIDSIM model. In particular, based on this new severity and vaccine protection data, we could assume a 3.2 reduction in infection severity for BA.1 or BA.2 with respect to Delta (*13*), and a 75% protection against infection, and a 95% protection against severe infection forms for vaccinated individuals with 2 doses and a booster (*14*).

Furthermore, epidemiologic data revealed that the serial interval, i.e., the distribution of the number of days between two infections (a proxy for the generation time) was lower for Omicron/BA.2 than for BA.1 (Figure 13 in [*16*]). This updated information was included in our second model.

As in the first model, we incorporated the frequency time series and growth advantage of BA.2 versus BA.1 estimated from the sequencing data (Figure 4, panel B in the main text) into the model.

Our analysis of the BA.2 wave included two steps:

First, from the French hospital admission data (available at https://www.data.gouv.fr/fr/datasets/donnees-hospitalieres-relatives-a-lepidemie-de-covid-19), we estimated the temporal reproduction number (Rt) of the epidemic using the EpiEstim package

in R (*6*). The estimated value for March 1, 2021, was R2022–03–01 = 0.83. We then compared this reference number to the Rt values predicted from our model based on the frequency and growth advantage of the Omicron variant. The results are shown in Figure 6, panel B.

Second, we explored two alternative scenarios over a medium time scale by varying the intensity of the control over the epidemic:

1. In an optimistic scenario, the control was assumed to remain strong and the Rt peaked at a value of 1.1.
2. In the pessimistic scenario, control measured were assumed to be strongly alleviated leading to an increase of Rt to 1.6.

As in the first models, these scenarios are not intended to predict the future but to explore an upper boundary regarding the potential consequences of an Omicron/BA.2 wave in France.

References

1. Chevin LM. On measuring selection in experimental evolution. Biol Lett. 2011;7:210–3. PubMed https://doi.org/10.1098/rsbl.2010.0580

2. Davies NG, Abbott S, Barnard RC, Jarvis CI, Kucharski AJ, Munday JD, et al.; CMMID COVID-19 Working Group; COVID-19 Genomics UK (COG-UK) Consortium. Estimated transmissibility and impact of SARS-CoV-2 lineage B.1.1.7 in England. Science. 2021;372:eabg3055. PubMed https://doi.org/10.1126/science.abg3055

3. Volz E, Mishra S, Chand M, Barrett JC, Johnson R, Geidelberg L, et al.; COVID-19 Genomics UK (COG-UK) consortium. Assessing transmissibility of SARS-CoV-2 lineage B.1.1.7 in England. Nature. 2021;593:266–9. PubMed https://doi.org/10.1038/s41586-021-03470-x

4. Alizon S, Haim-Boukobza S, Foulongne V, Verdurme L, Trombert-Paolantoni S, Lecorche E, et al. Rapid spread of the SARS-CoV-2 Delta variant in some French regions, June 2021. Euro Surveill. 2021;26:2100573. PubMed https://doi.org/10.2807/1560-7917.ES.2021.26.28.2100573

5. Nishiura H, Linton NM, Akhmetzhanov AR. Serial interval of novel coronavirus (COVID-19) infections. Int J Infect Dis. 2020;93:284–6. PubMed https://doi.org/10.1016/j.ijid.2020.02.060

6. Cori A, Ferguson NM, Fraser C, Cauchemez S. A new framework and software to estimate time-varying reproduction numbers during epidemics. Am J Epidemiol. 2013;178:1505–12. PubMed https://doi.org/10.1093/aje/kwt133

7. Hay JA, Kennedy-Shaffer L, Kanjilal S, Lennon NJ, Gabriel SB, Lipsitch M, et al. Estimating epidemiologic dynamics from cross-sectional viral load distributions. Science. 2021;373:eabh0635. PubMed https://doi.org/10.1126/science.abh0635

8. Alizon S, Selinger C, Sofonea MT, Haim-Boukobza S, Giannoli JM, Ninove L, et al.; SFM COVID-19 study group. Epidemiological and clinical insights from SARS-CoV-2 RT-PCR crossing threshold values, France, January to November 2020. Euro Surveill. 2022;27:2100406. PubMed https://doi.org/10.2807/1560-7917.ES.2022.27.6.2100406

9. Hall VJ, Foulkes S, Charlett A, Atti A, Monk EJM, Simmons R, et al.; SIREN Study Group. SARS-CoV-2 infection rates of antibody-positive compared with antibody-negative health-care workers in England: a large, multicentre, prospective cohort study (SIREN). Lancet. 2021;397:1459–69. PubMed https://doi.org/10.1016/S0140-6736(21)00675-9

10. Sofonea MT, Reyné B, Elie B, Djidjou-Demasse R, Selinger C, Michalakis Y, et al. Memory is key in capturing COVID-19 epidemiological dynamics. Epidemics. 2021;35:100459. PubMed https://doi.org/10.1016/j.epidem.2021.100459

11. O'Driscoll M, Ribeiro Dos Santos G, Wang L, Cummings DAT, Azman AS, Paireau J, et al. Age-specific mortality and immunity patterns of SARS-CoV-2. Nature. 2021;590:140–5. PubMed https://doi.org/10.1038/s41586-020-2918-0

12. Salje H, Tran Kiem C, Lefrancq N, Courtejoie N, Bosetti P, Paireau J, et al. Estimating the burden of SARS-CoV-2 in France. Science. 2020;369:208–11. PubMed https://doi.org/10.1126/science.abc3517

13. Nyberg T, Ferguson NM, Nash SG, Webster HH, Flaxman S, Andrews N, et al.; COVID-19 Genomics UK (COG-UK) consortium. Comparative analysis of the risks of hospitalisation and death associated with SARS-CoV-2 omicron (B.1.1.529) and delta (B.1.617.2) variants in England: a cohort study. Lancet. 2022;399:1303–12. PubMed https://doi.org/10.1016/S0140-6736(22)00462-7

14. UK Health Security Agency. COVID-19 vaccine surveillance report, week 10. 2022 Mar 10 [cited 2022 May 6]. https://assets.publishing.service.gov.uk/government/uploads/system/uploads/attachment_data/file/1060787/Vaccine_surveillance_report_-_week_10.pdf

15. Smith TP, Flaxman S, Gallinat AS, Kinosian SP, Stemkovski M, Unwin HJT, et al. Temperature and population density influence SARS-CoV-2 transmission in the absence of nonpharmaceutical interventions. Proc Natl Acad Sci U S A. 2021;118:e2019284118. PubMed https://doi.org/10.1073/pnas.2019284118

16. UK Health Security Agency. SARS-CoV-2 variants of concern and variants under investigation in Technical briefing 36. 2022 Feb 11 [cited 2022 May 6]. https://assets.publishing.service.gov.uk/government/uploads/system/uploads/attachment_data/file/1056487/Technical-Briefing-36-22.02.22.pdf

Appendix 1 Table 1. Type II Anova model output for the linear model analyzing variations in Ct values*

Variable	Sum Sq	Df	F value
age	14429	1	1290.9
location_sampling	114	1	10.2
date	3670	1	328.3
region2	40363	12	300.9
variant	5084	3	151.6
date:region	6877	12	51.3
Residuals	1526899	136605	

*<0.001 indicates significance values lower than 0.001.

Appendix 1 Table 2. Output of the linear model analyzing variations in Ct values*

Dependent variable	Ct
age	−0.329***
	(0.009)
location_sampling:hospital	−0.175***
	(0.055)
date_scale	0.284***
	(0.022)
variant:A0B9C0D0	0.304***
	(0.087)
variant:A0B9C1D0	−0.746***
	(0.036)
variant:A0B9C1D1	−0.084
	(0.273)
region:Auvergne-Rhône-Alpes	−0.375**
	(0.160)
region:Bourgogne-Franche-Comté	−1.066***
	(0.060)
region:Bretagne	0.424***
	(0.059)
region:Centre-Val de Loire	0.620***
	(0.052)
region:Corse	−0.852***
	(0.050)
region:Grand Est	−1.289***
	(0.051)
region:Hauts-de-France	−0.943***
	(0.034)
region:Normandie	0.161***
	(0.035)
region:Nouvelle-Aquitaine	−1.026***
	(0.042)
region:Occitanie	−0.648***
	(0.047)
region:Pays de la Loire	−0.436***
	(0.111)

Dependent variable	Ct
region:Provence-Alpes-Côte d’Azur	−0.452***
	(0.033)
date*region:Auvergne-Rhône-Alpes	−0.303*
	(0.158)
date*region:Bourgogne-Franche-Comté	−0.338***
	(0.060)
date*region:Bretagne	0.647***
	(0.051)
date*region:Centre-Val de Loire	0.258***
	(0.051)
date*region:Corse	0.126***
	(0.047)
date*region:Grand Est	−0.466***
	(0.056)
date*region:Hauts-de-France	−0.321***
	(0.033)
date*region:Normandie	−0.048
	(0.036)
date*region:Nouvelle-Aquitaine	−0.329***
	(0.043)
date*region:Occitanie	−0.243***
	(0.046)
date*region:Pays de la Loire	−0.626***
	(0.107)
date*region:Provence-Alpes-Côte d’Azur	−0.102***
	(0.033)
Constant	22.669***
	(0.025)
Observations	136,636
R2	0.046
Adjusted R2	0.045
Residual Std. Error	3.343 (df = 136605)
F Statistic	217.335*** (df = 30; 136605)

*For each covariate, we show the estimated value of the effect and its standard deviation in parentheses. Significance level are shown using the following code: *p < 0.1, **p < 0.05 and ***p < 0.01. The table was formatted with the stargazer R package.

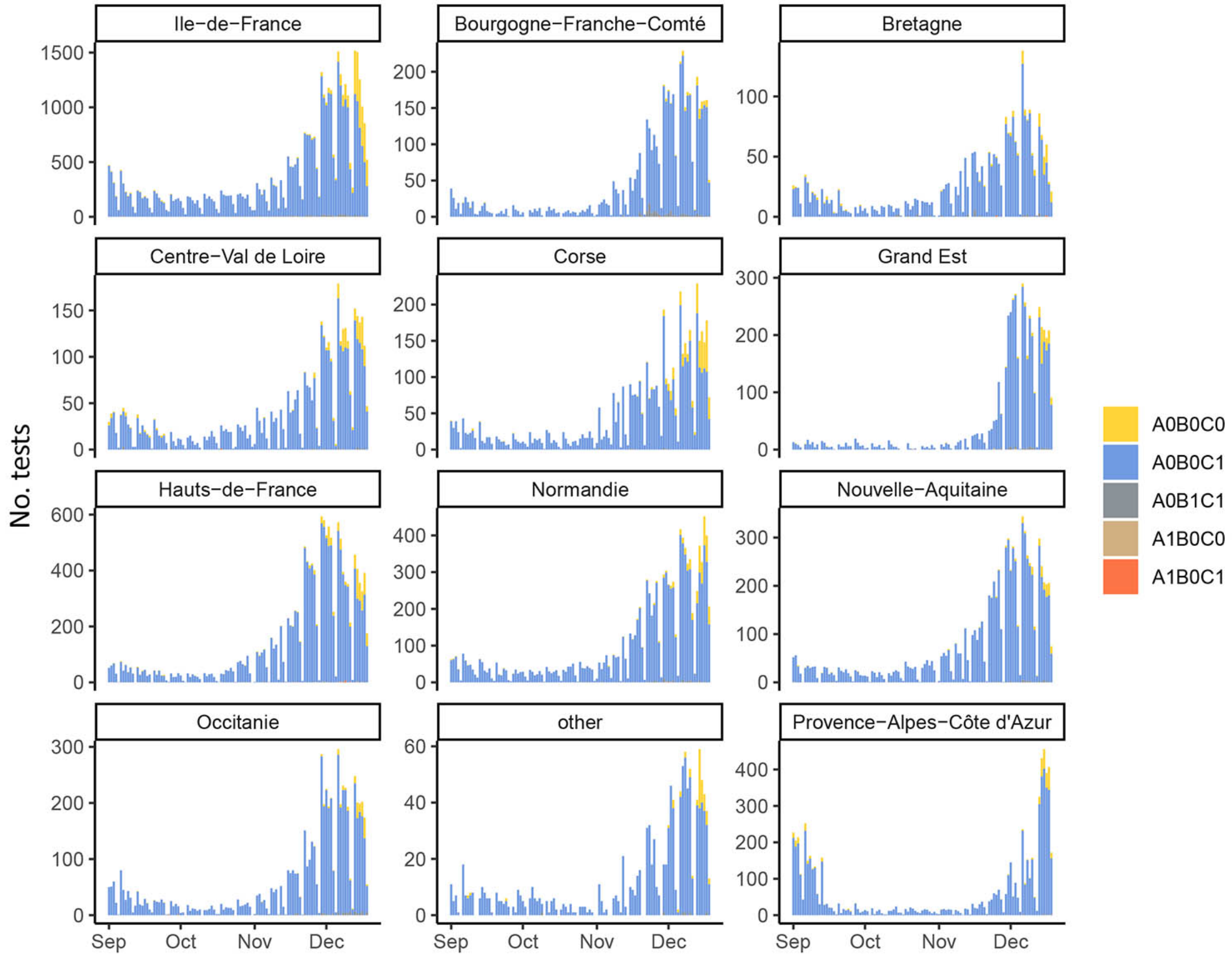

Appendix 1 Figure 1. Variant-specific screening test incidence data per French region. The color indicates the test result. Regions with too few tests are pooled in the "other" category. Notice that the epidemic was declining until mid-October 2021. Furthermore, the proportion of A0B0C0 tests varies across regions.

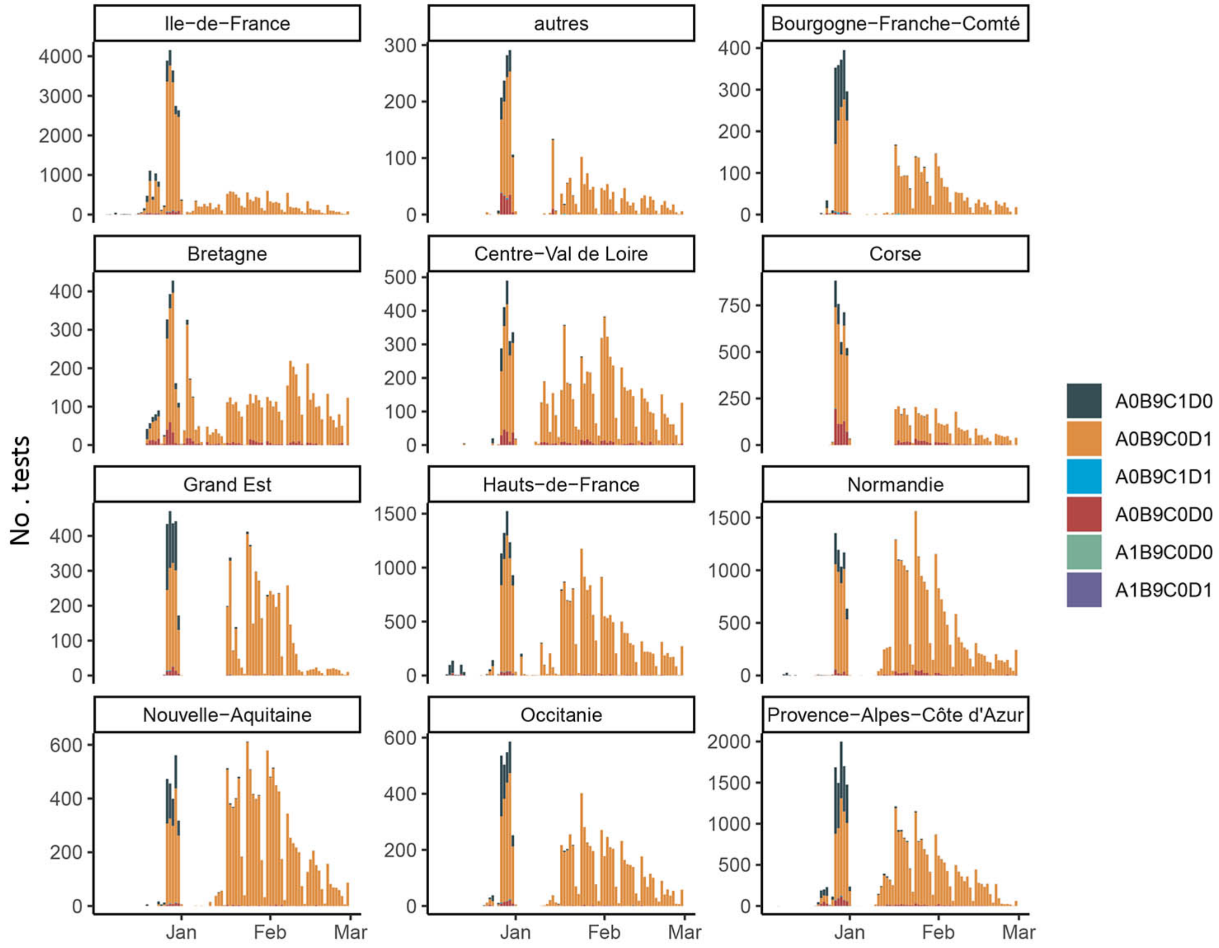

Appendix 1 Figure 2. Variant-specific screening test incidence data per French region for the ID solutions Revolution test. The color indicates the test result. Regions with too few tests are pooled in the “other” category.

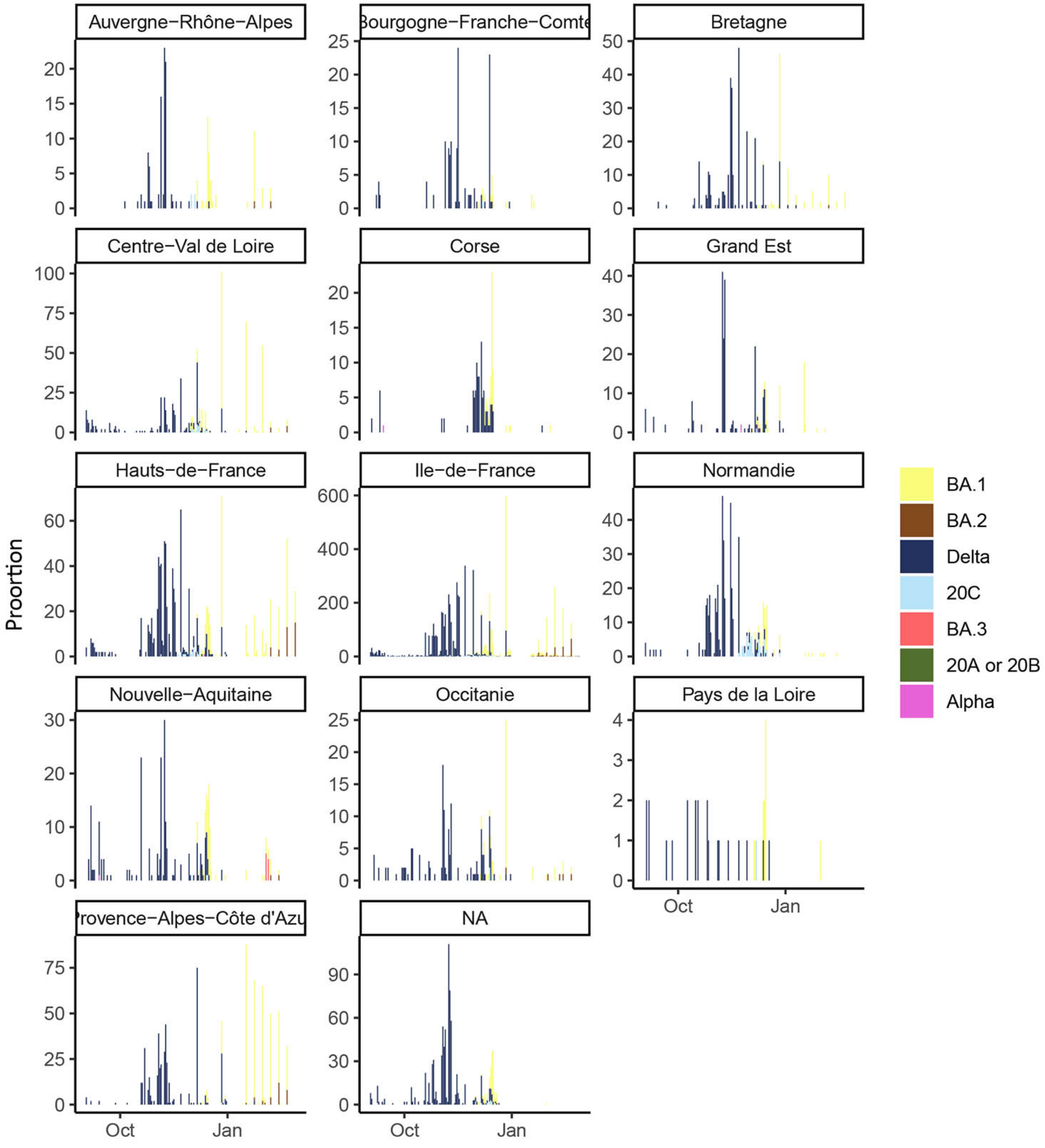

Appendix 1 Figure 3. Lineage-specific incidence data per French region using whole genome sequencing. Samples with missing regions are in the "NA" category.

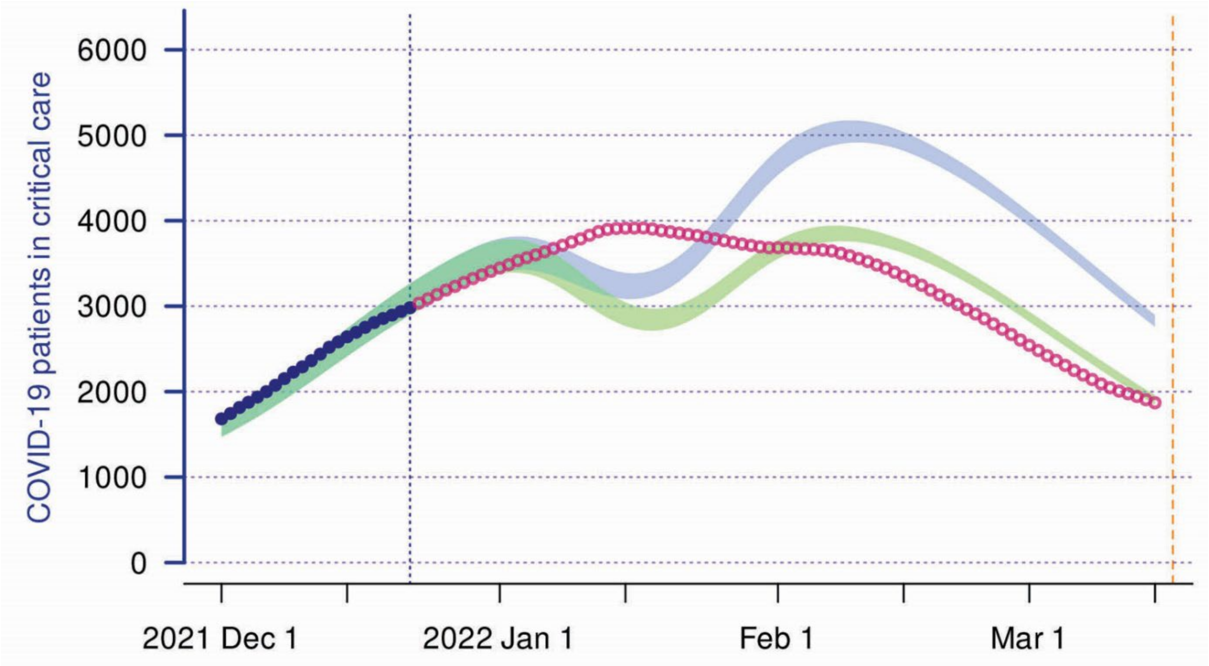

Appendix 1 Figure 4. Epidemiologic modeling of scenarios for French national critical care unit (CCU) occupancy from December 2021 to Mid-March 22 with different Omicron virulence and immune evasion properties. The vertical blue line indicates the day the simulations were performed (22 Dec 2021), using solely epidemiologic, hospital, and virological data available to that date. In particular, the dark blue dots indicate the nationwide number critical care beds occupied by COVID-19 patients included for the model inference. The yellow vertical bar shows the date the figure was made. The red circles correspond to the data observed after the computation of the projections (data from Santé publique France). The shaded envelope the 95% range of the optimistic (green) and pessimistic (blue) scenarios according to the estimates available on Dec 22 2021. As expected, the model captures the CCU dynamics during the first 2 weeks. The underestimation of the CCU occupancy in mid-Jan is related to the (overoptimistic) assumption that the Delta epidemic was under control when the model was made. This led to a merging between the Delta and the Omicron/BA.1 epidemic peaks in CCU on January 24, 2022. The ‘optimistic’ scenario (with low Omicron virulence and high vaccine protection) provided an accurate lower boundary for CCU occupancy until the end of January 2022. See the main text and the supplementary methods for more details about the model and its assumptions.
